# Identification of Biomarkers That Modulate Osteogenic Differentiation in Mesenchymal Stem Cells Related to Inflammation and Immunity: A Bioinformatics-Based Comprehensive Study

**DOI:** 10.3390/ph15091094

**Published:** 2022-08-31

**Authors:** Ziyi Feng, Xin Su, Ting Wang, Shu Guo

**Affiliations:** Department of Plastic Surgery, The First Hospital of China Medical University, No. 155, Nanjing North Street, Heping District, Shenyang 110002, China

**Keywords:** bioinformatics, osteogenic differentiation, mesenchymal stem cells, biomarker, immunity, inflammation

## Abstract

Background: Inducing mesenchymal stem cells (MSCs) osteogenesis may be beneficial in a number of clinical applications. The aim of this study is to identify key novel biomarkers of this process and to analyze the possible regulatory effects on inflammation and immunity. Results: Seven datasets (GSE159137, GSE159138, GSE114117, GSE88865, GSE153829, GSE63754, GSE73087) were obtained from the Gene Expression Omnibus database and were assigned to either the training or the validation dataset. The least absolute shrinkage and selection operator (LASSO) logistic regression model was applied to the training data to select biomarkers of osteogenesis, which were then confirmed using the validation dataset. FK506 binding protein 5 (FKBP5), insulin-like growth factor binding protein (IGFBP2), prostaglandin E receptor 2 (PTGER2), SAM domain and HD domain-containing protein 1 (SAMHD1), and transmembrane tetratricopeptide 1 (TMTC1) were highlighted as potential biomarkers. In addition, the differential expressions of immunity and inflammation-related genes were examined and their correlations with the five identified biomarkers were analyzed. The results from performing RT-qPCR and Western blots confirmed that the levels of each of these biomarkers were all significantly increased following osteogenic differentiation of MSCs. Conclusions: Our results identify five biomarkers related to MSCs osteogenesis and allow us to identify their potential roles in immunoregulation and inflammation. Each biomarker was verified by in vitro experiments.

## 1. Introduction

The reparation of substantial bone defects in order to restore the anatomy, function, and appearance of a damaged area continues to be a major challenge for clinicians. However, recent stem cell-based therapy (SCBT) has shown significant potential for the treatment of bone defects, including severe damage [1,2].

Mesenchymal stem cells (MSCs) are pluripotent stem cells that can be isolated from virtually all mesodermal tissues including bone marrow, adipose tissue, cord blood, umbilical cord, placenta, menstrual fluid, and dental pulp [3,4]. The International Society for Cellular Therapy (ISCT) has defined minimum criteria for characterizing MSCs that include: fibroblast-like morphology; expression of mesodermal markers (e.g., CD90, CD105, and CD73); lack of hematopoietic marker expression (e.g., CD45, CD34, CD14) and the ability to differentiate into adipocytes, chondrocytes, and osteoblasts [5]. Thus, bone regeneration represents a suitable, though often challenging, target to test the specific application of stem cell therapy [6,7]. In addition to the effective repair of tissue defects, MSCs can also be used to treat various immune system diseases through immunomodulation. Dependent on the microenvironment, MSCs exhibit different regulatory properties relating to both immune and inflammatory responses via direct immune cell contact and the production of inflammatory regulatory molecules (namely, the paracrine effects) [8,9]. Through their unique influence on the immune system, MSCs have the capability to both up- and down-regulate inflammatory processes. Up-regulation mainly occurs in response to “danger signals” and involves the release of anti-inflammatory mediators through activated Toll-like receptor (TLR) ligands (such as TLR9, TLR7, and TLR2), whereas down-regulation involves the expression of the immune suppressors PD-L1 and PD-L2 due to the stimulation of interferon gamma (IFN-γ) [10]. MSCs also have various roles in coordinating the migration, proliferation, and activation of immune cells, depending on the stage of osteogenesis and the type of immune cells [11]. Changes in pro-inflammatory and anti-inflammatory cytokines found in a micro-environment cause MSCs to secrete prostaglandin E2 (PGE2), transforming the growth factor beta (TGF-β) and the histocompatibility locus antigen-G (HLA-G). These secretions consequently induce the formation of regulatory T cells, suppress neutrophil migration, and most importantly, play a role in immune inhibition through monocyte/macrophage regulation [12,13,14]. Therefore, identifying biomarkers of osteogenic differentiation that can be clinically evaluated is vital to the establishment of relevant treatment strategies for bone defects. Furthermore, exploring the potential roles of immunomodulation and inflammatory regulation involved in MSCs osteogenic differentiation is crucial for discovering novel therapeutic strategies and improving bone remodeling [15].

**Figure 1:**
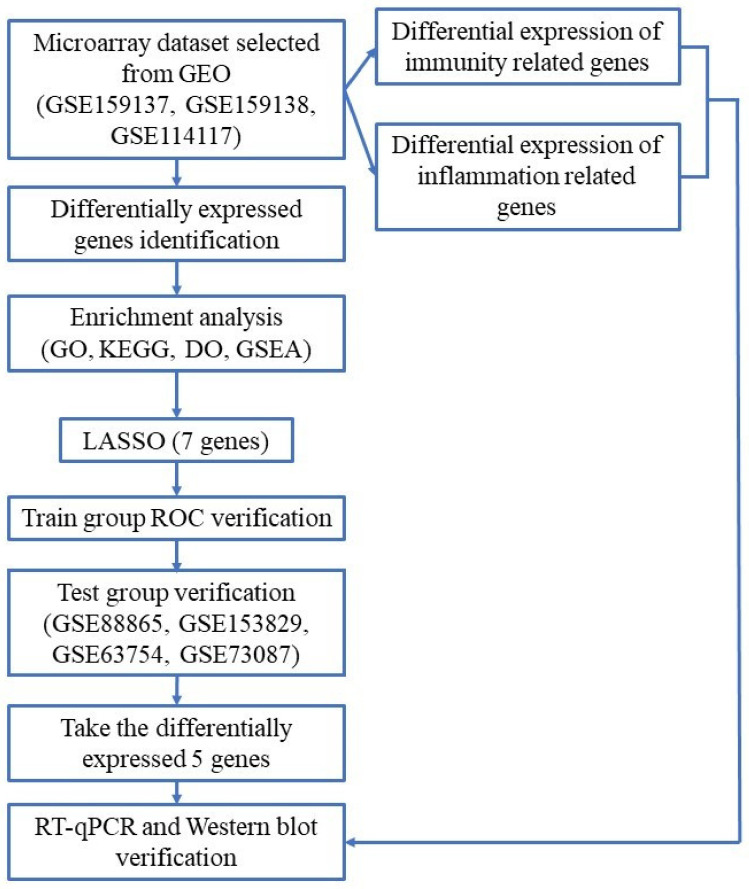
Workflow chart of data generation and analysis.

This study utilizes a comprehensive strategy to determine biomarkers associated with osteogenic differentiation of MSCs and further investigates the relationship between these biomarkers and the genes involved in the immunoregulation/inflammation that occurs during this process. The objective of our research is to provide a platform for further exploration of significant genes, biochemical pathways, as well as vital bioactive molecules affecting osteogenic induction of mesenchymal stem cells. The study flowchart is presented in Figure 1.

## 2. Results

### 2.1. Identification of DEGs 

**Figure 2:**
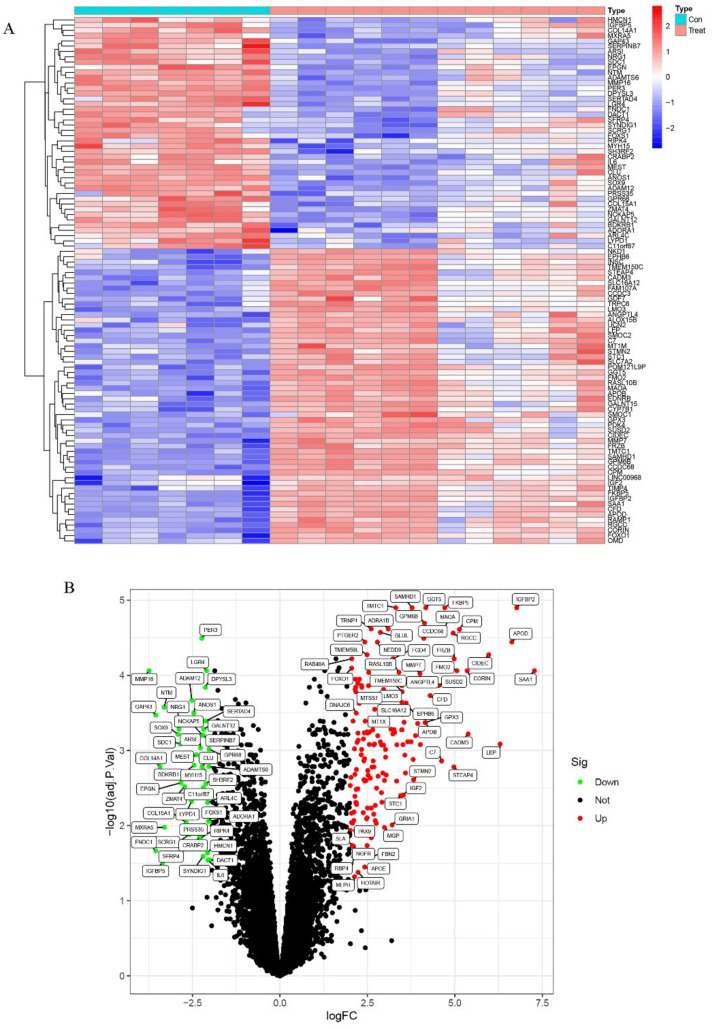
The expression characteristics of genes in osteogenic−induced mesenchymal stem cells (treat group) and uninduced mesenchymal stem cells (control group). (**A**) Heatmap presented the overall expression of genes with FDR < 0.05 in treat and control group. (**B**) Volcano plot visualized the upregulated (red) and downregulated (blue) genes in treat and control group.

Firstly, the integrated training datasets (GSE159137, GSE159138, and GSE114117) were screened for differentially expressed genes (DEGs). A total of 7 uninduced MSCs samples and 12 osteogenic-induced MSCs samples were included in this analysis. There were significant differences between the control (uninduced) and the induced groups. Overall, 200 DEGs were observed in the osteogenic induction group and the control group, comprising 156 up-regulated and 44 down-regulated genes. These DEGs are displayed as a heatmap (Figure 2A) and volcano plots (Figure 2B).

### 2.2. Functional Enrichment Analyses of DEGs

**Figure 3:**
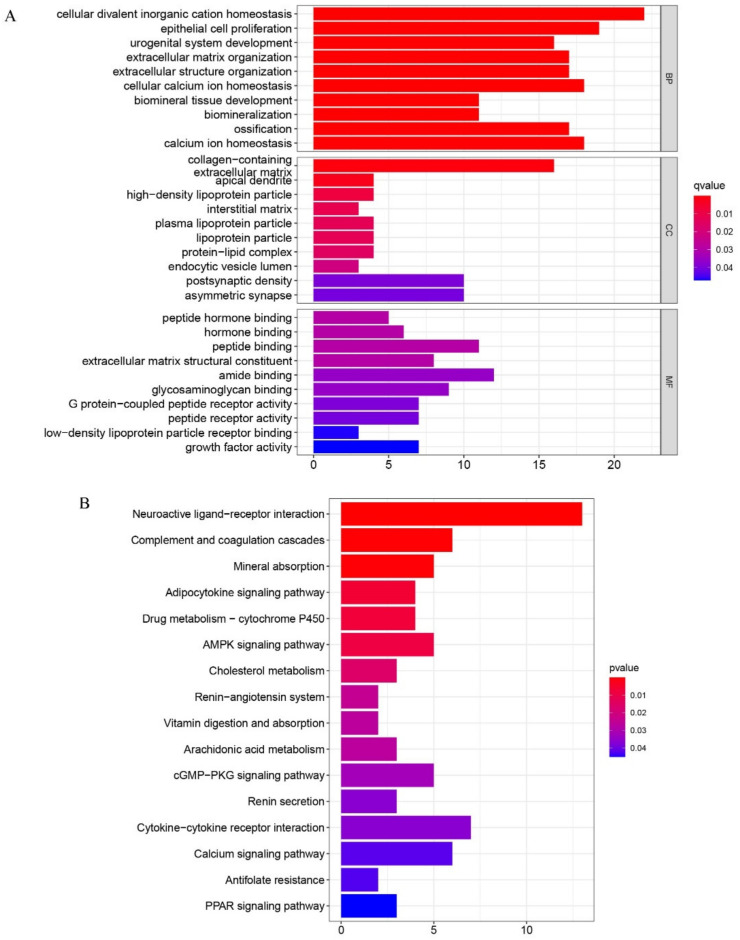
(**A**) Gene Ontology (GO) analyses were conducted to predict the potential function of the differentially expressing genes between the treat and the control group including cellular component (CC), molecular function (MF), biological process (BP), respectively. (**B**) Kyoto Encyclopedia of Genes and Genomes (KEGG) predicted the potential pathways regarding the differentially genes between the treat and the control group.

**Figure 4:**
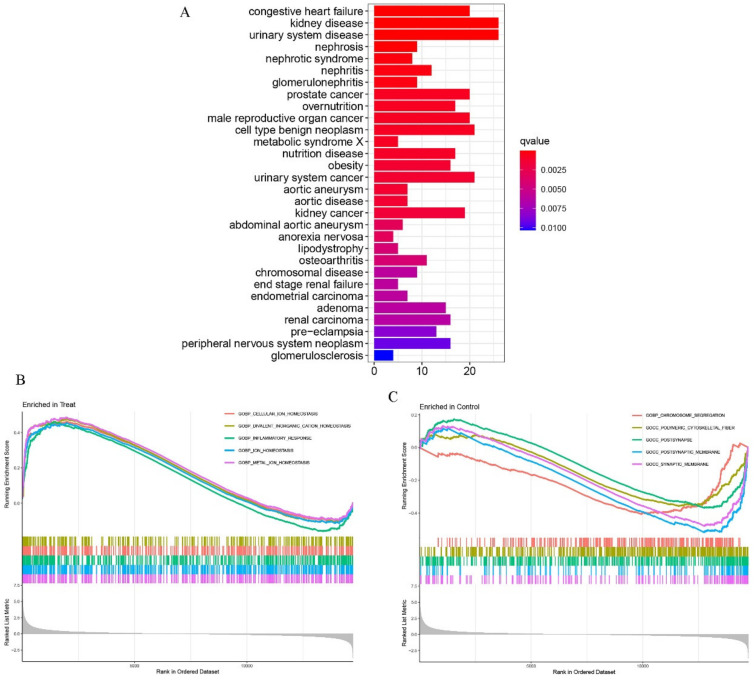
(**A**) Disease ontology (DO) analyses were conducted to predict the potential related diseases of the differentially expressing genes between the treat and the control group. (**B**,**C**) GSEA showed that the top 5 signal pathways were most related to treat (**B**) and control group (**C**).

To demonstrate the functional groups of the DEGs, gene set enrichment analysis (GSEA) was conducted using the “ClusterProfiler” package. Gene Ontology (GO) analysis showed that the principal biological functions of the enriched DEGs were cellular divalent inorganic cation homeostasis, collagen-containing extracellular matrix, and amide binding, all of which are involved in the cell growth process (Figure 3A). To further illustrate the bio-function of these DEGs, KEGG (Kyoto Encyclopedia of Genes and Genomes) analysis was performed. The results demonstrate that neuroactive ligand–receptor interaction, as well as cytokine–cytokine receptor interaction, were the most significant processes occurring during the MSC osteogenic differentiation process (Figure 3B). DO analysis revealed that these DEGs may also be important for understanding the pathogenesis of both kidney and urinary system diseases (Figure 4A).

Moreover, GSEA was used to explore the variation in molecular pathways (and their underlying mechanisms) exhibited in the osteogenic induced and control groups (Figure 4B). The following were shown to be active in the former group: cellular ion homeostasis, divalent inorganic cation homeostasis, and inflammatory response, whereas chromosome segregation, polymeric cytoskeletal fiber, post-synapse, post-synaptic membrane, and synaptic membrane were identified in the latter (Figure 4C). Therefore, we were able to link DEGs to biological functions.

### 2.3. Verification of the Biomarkers

**Figure 5:**
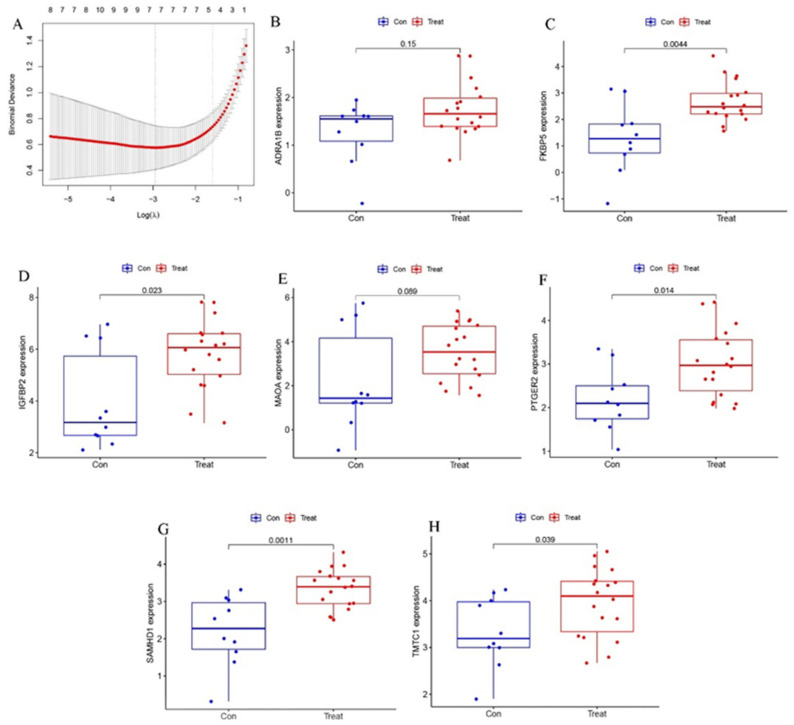
(**A**) The least absolute shrinkage and selection operator (LASSO) logistic regression algorithm was used to retain the most predictive features. (**B**−**H**) The differential expression of ADRA1B (**B**), FKBP5 (**C**), IGFBP2 (**D**), MAOA (**E**), PTGER2 (**F**), SAMHD1 (**G**) and TMTC1 (**H**) in test group.

LASSO regression was used to calculate the importance score for each gene via the “glmnet” package, in order to identify potential biomarkers. A total of seven genes were identified from all DEGs (Figure 5A). These genes encode proteins deemed suitable for use as biomarkers, including adrenoceptor alpha 1B (ADRA1B), FKBP prolyl isomerase 5 (FKBP5), insulin-like growth factor binding protein 2 (IGFBP2), monoamine oxidase A (MAOA), prostaglandin E receptor 2 (PTGER2), SAM and HD domain-containing deoxynucleoside triphosphate triphosphohydrolase 1 (SAMHD1), and transmembrane O-mannosyltransferase targeting cadherins 1 (TMTC1).

**Figure 6:**
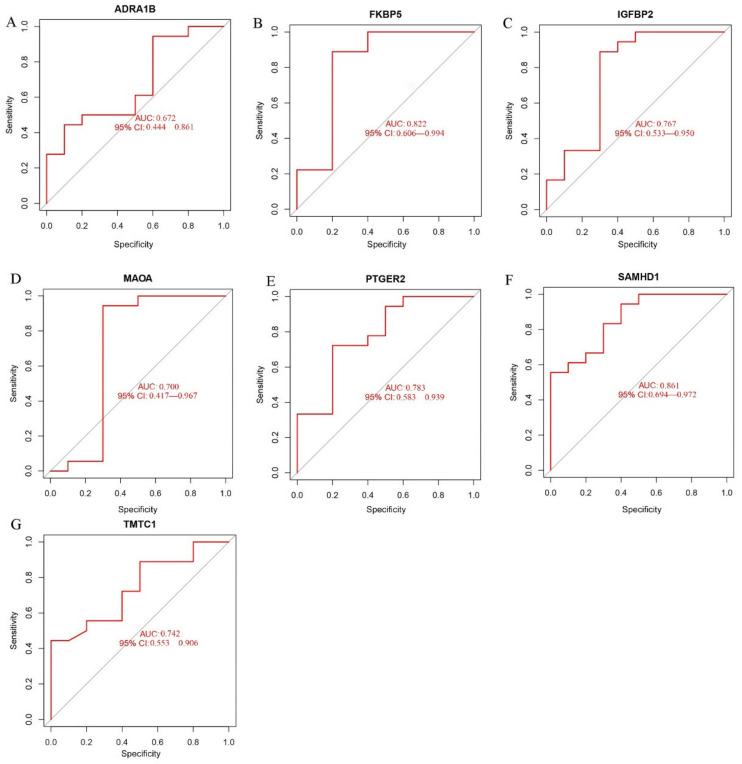
(**A**−**G**) Receiver operating characteristic (ROC) curves of ADRA1B (**A**), FKBP5 (**B**), IGFBP2 (**C**), MAOA (**D**), PTGER2 (**E**), SAMHD1 (**F**) and TMTC1 (**G**) in test group.

To assess the accuracy of these candidate biomarkers, we used four verification datasets (GSE88865, GSE153829, GSE63754, GSE73087) to validate efficacy. Of the seven putative biomarkers, five were verified (including FKBP5, IGFBP2, PTGER2, SAMHD1, and TMTC1) as having significant elevations in expression in the osteogenic induced groups, compared with the control group (Figure 5B–H). However, this was not the case for ADRA1B and MAOA, thus they were discarded and not subjected to further validation as biomarkers. ROC curves were used to further determine the diagnostic value of the remaining five biomarkers in terms of osteogenic induction of MSCs. When the validation datasets were analyzed, all five selected biomarkers had an Area Under Curve (AUC) > 0.7, confirming their ability to diagnose MSCs osteogenic differentiation with excellent specificity and sensitivity (Figure 6). It is worth noting that SAMHD1 showed the greatest potential, with the highest AUC (0.861).

### 2.4. Identification of DEGs Related to Immunity and Inflammation

**Figure 7:**
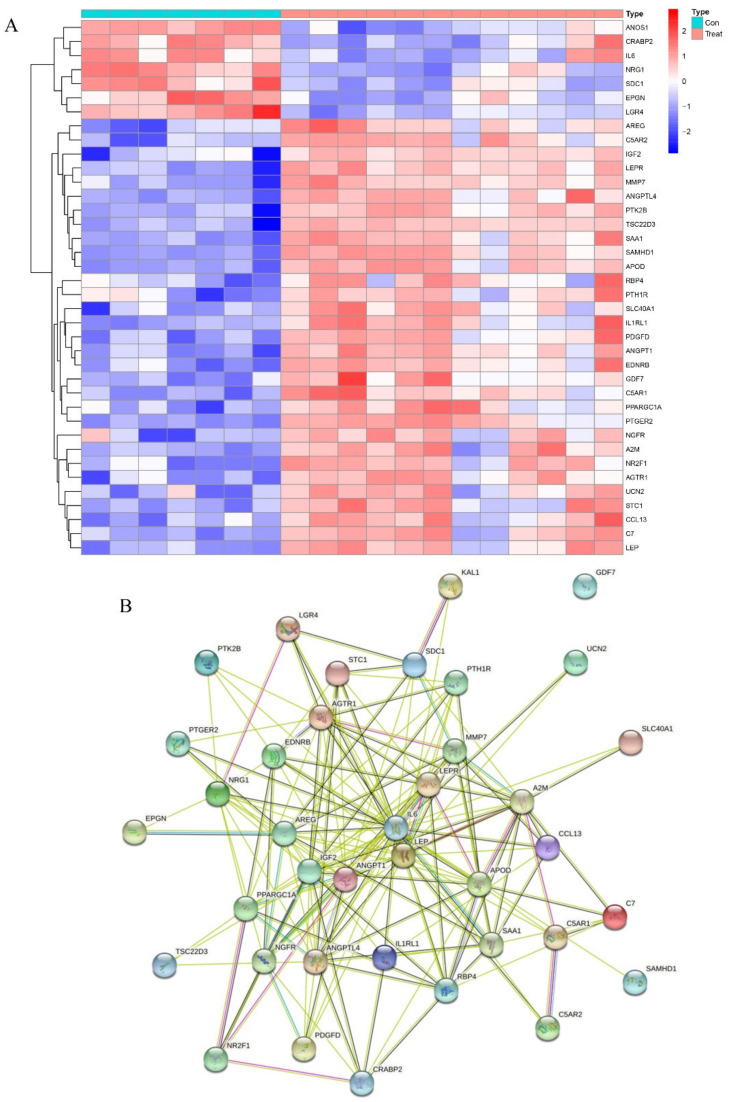
The expression characteristics of immunity related regulators. (**A**) Heatmaps presented the expression of inflammation related regulators in treat and control group. (**B**) The STRING Protein−Protein Interaction Networks of immunity related regulators.

**Figure 8:**
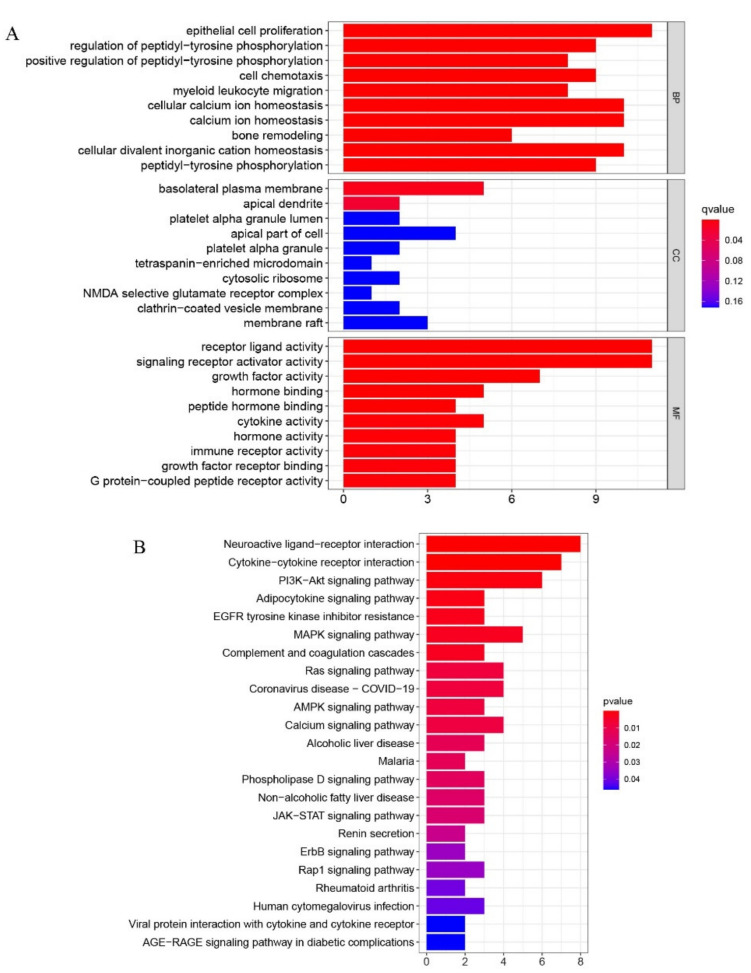
(**A**) Gene Ontology (GO) analyses were conducted to predict the potential function of the differentially expressing immunity related genes between the treat and the control group, including cellular component (CC), molecular function (MF), biological process (BP), respectively. (**B**) Kyoto Encyclopedia of Genes and Genomes (KEGG) predicted potential pathways regarding the differentially immunity related genes between the treat and the control group.

Heatmaps comparing the osteogenic induced and control groups revealed 38 differentially expressed immunity-related genes, with FDR *q*-value < 0.05, that have a regulatory role (Figure 7A). Of these, 31 were up-regulated and the remaining 7 were down-regulated. To verify the relationships of these immunity-related regulators at a protein level, we drew the protein–protein interaction (PPI) network (Figure 7B). We also performed functional enrichment analysis using the STRING database, for a more in-depth analysis of protein functions. The overall consensus of these analyses indicated that, out of all the genes studied, IL6, LEP, and ANGPT1 had the most interactions. GO and KEGG analyses were performed on immunity-related DEGS to determine which pathways and associated functions were likely to be encoded by these genes. The GO (Figure 8A) results revealed that the differentially expressed immunity-related regulators were predominantly concentrated in the biological process (BP) of epithelial cell proliferation, basolateral plasma membrane, receptor–ligand activity in cellular components (CC), and the molecular function (MF), signaling receptor activator activity. In KEGG (Figure 8B), DEGs are concentrated in the neuroactive ligand–receptor interaction, as well as the cytokine–cytokine receptor interaction.

**Figure 9:**
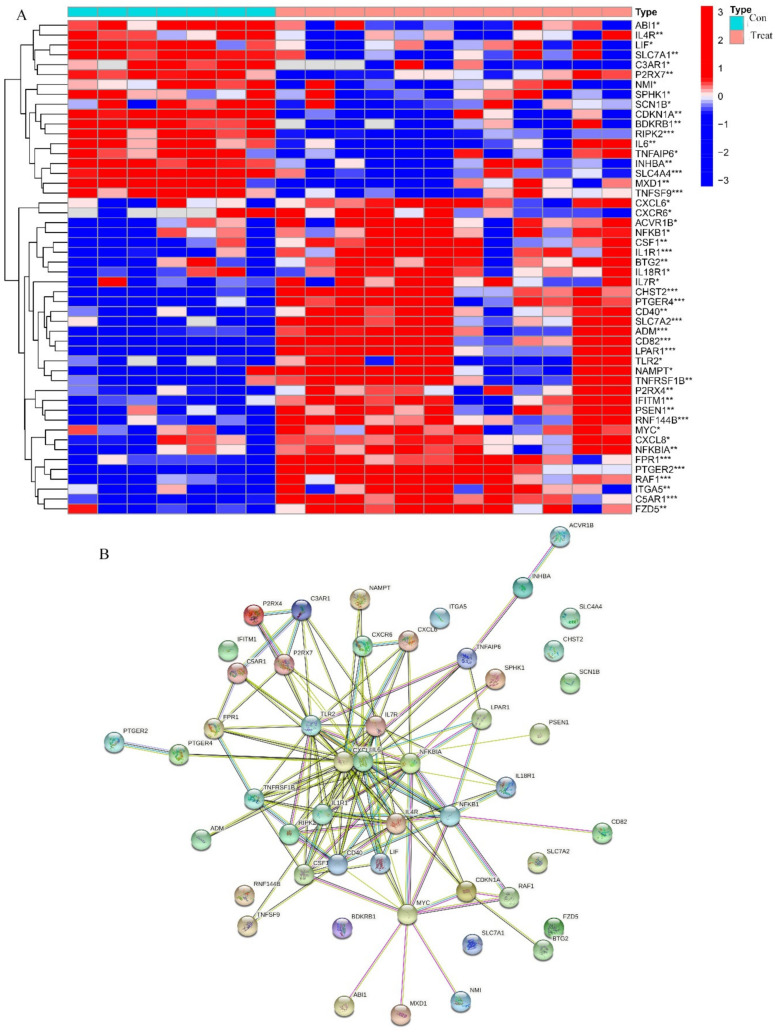
The expression characteristics of inflammation related regulators. (**A**) Heatmaps presented the expression of inflammation related regulators in treat and control group. *p* < 0.05 (“*”), *p* < 0.01 (“**”) and *p* < 0.001 (“***”). (**B**) The STRING Protein−Protein Interaction Networks of inflammation related regulators.

**Figure 10:**
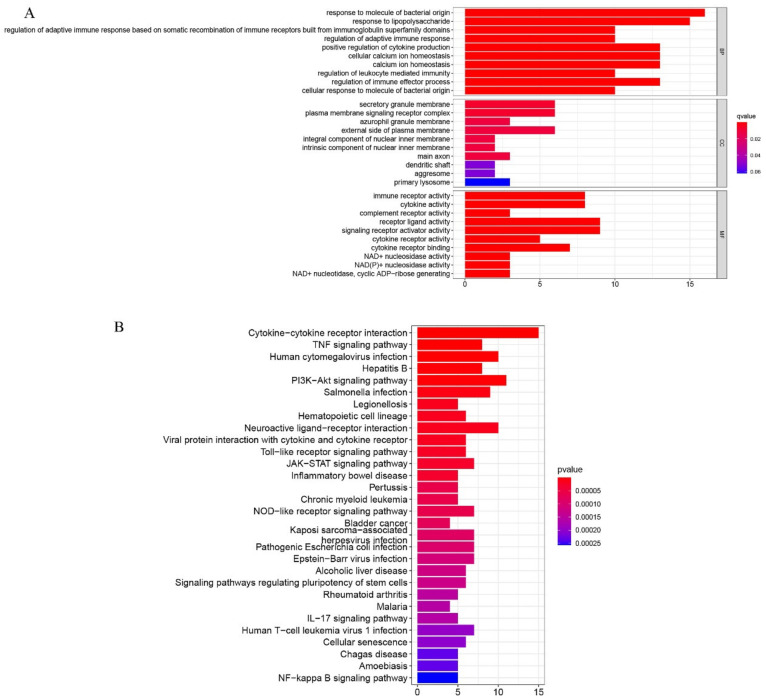
(**A**) Gene Ontology (GO) analyses were conducted to predict the potential function of the differentially expressing inflammation related genes between the treat and the control group, including cellular component (CC), molecular function (MF), biological process (BP), respectively. (**B**) Kyoto Encyclopedia of Genes and Genomes (KEGG) predicted potential pathways regarding the differentially inflammation related genes between the treat and the control group.

Again, comparing the osteogenic induced and control groups, we screened differentially expressed inflammation-related regulators using the cut-off, *p*-value < 0.05. The results, which showed 32 up-regulated and 18 down-regulated genes, are presented as heatmaps (Figure 9A). The interactions, as well as the expression number, of these inflammation-related regulators, are shown in Figure 9B. CXCL8 and IL6 were treated as the central genes and the interactions suggested that the DEGs were functionally associated as well. GO and KEGG enrichment analyses identified the response to molecules of bacterial origin and the response to lipopolysaccharides in BP, secretory granule membrane, and plasma membrane, signaling receptor complex in CC, receptor–ligand activity and signaling receptor activator activity in MF, while cytokine–cytokine receptor interaction was the most important function and pathway of the differentially expressing inflammation-related regulators according to the enrichment scores in KEGG (Figure 10).

### 2.5. Potential Roles of Hub Biomarkers in Immunity and Inflammation

**Figure 11:**
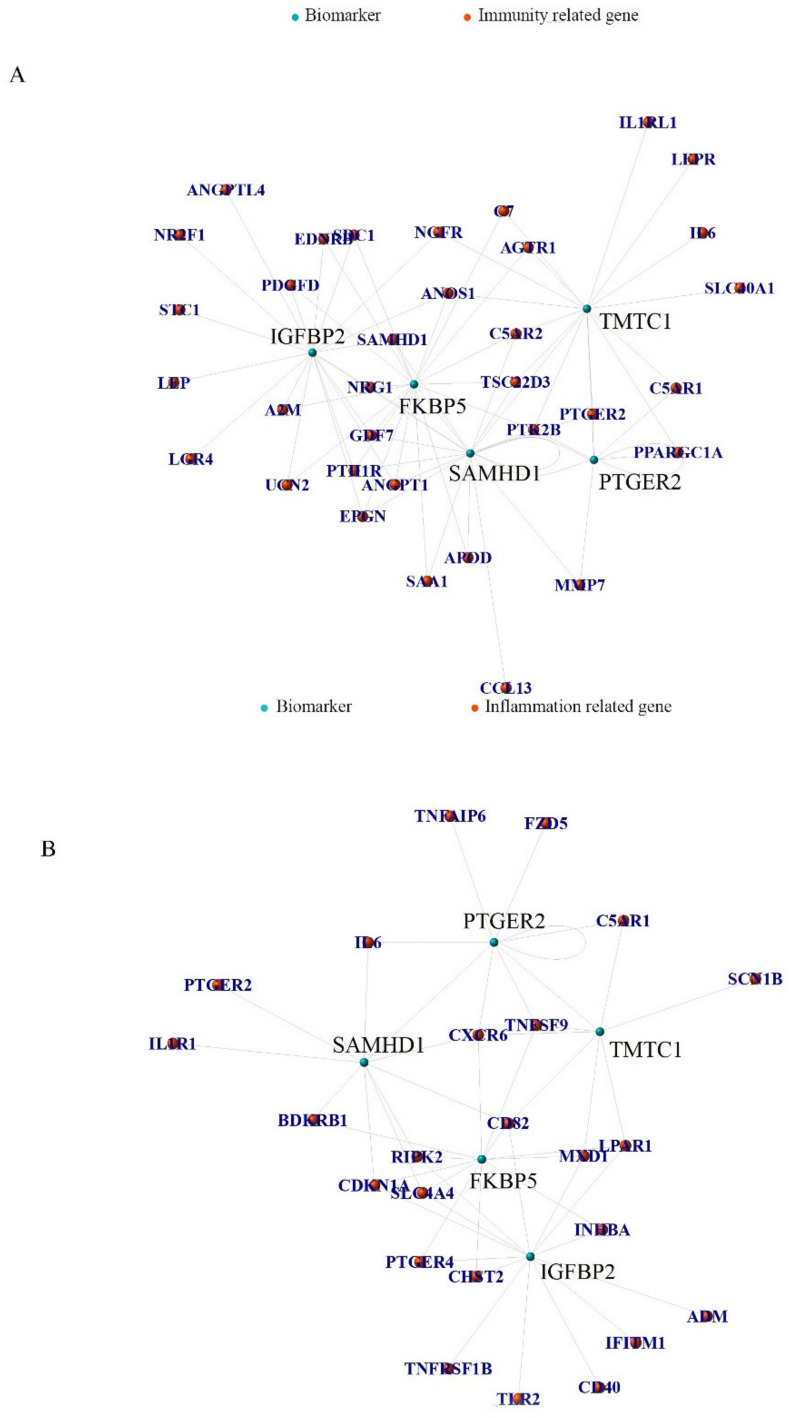
(**A**) Relationship between differentially expressed immunity related genes and selected biomarkers. (**B**) Relationship between differentially expressed inflammation related genes and selected biomarkers.

Using RNA-seq to profile the transcriptome, we investigate the association between differentially expressed immunity-related genes and selected biomarkers (Figure 11A), as well as the relationship between differentially expressed inflammation-related genes and the five hub biomarkers (Figure 11B). The correlation coefficients and positive and negative regulatory relationships between the biomarkers are shown in Supplementary Tables S1 and S2. During stem cell osteogenic induction, the identified biomarkers of this process showed a strong correlation with immunity and inflammation. These associations provide a platform for further research into the relationship between osteogenesis-related biomarkers of MSCs and immunity/inflammatory regulation.

### 2.6. Validation of Hub Biomarkers

**Figure 12:**
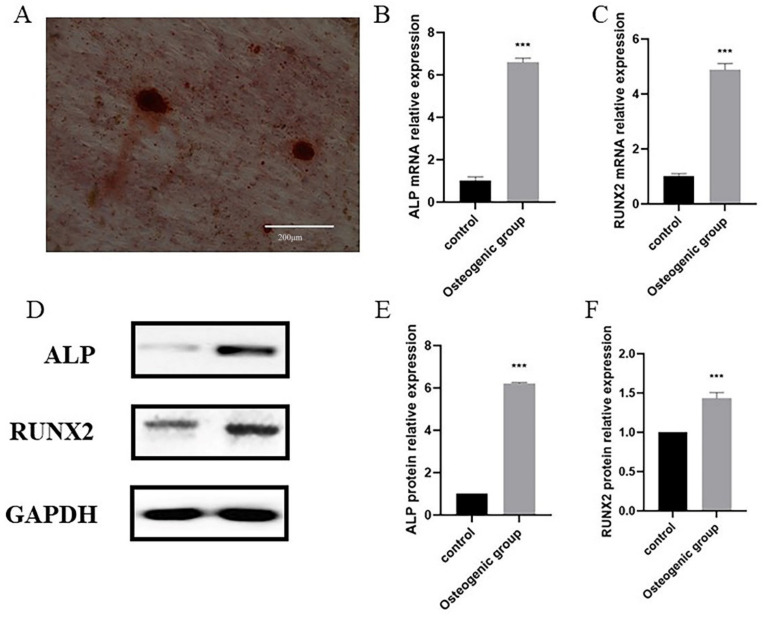
(**A**) The osteogenic induced of ADSCs to differentiate into osteoblasts confirmed by Alizarin Red S. (**B**−**C**) The expression of ALP (**B**) and RUNX2 (**C**) quantified by qPCR in the uninduced and the osteogenic induced of ADSCs. (**D**) The expression of the ALP and RUNX2 analyzed by Western blot. (**E**−**F**) Relative intensity analyses of Western blot results of ALP (**E**), RUNX2 (**F**). *p* < 0.001 (“***”).

**Figure 13:**
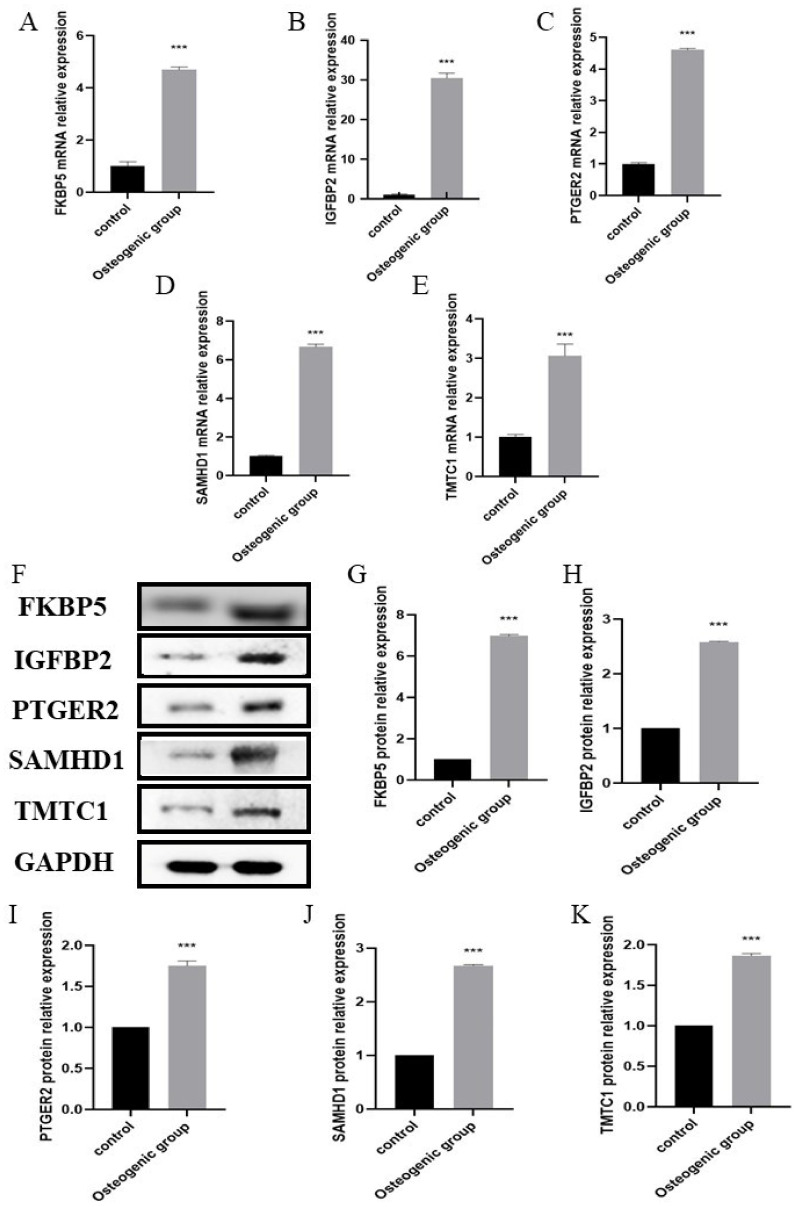
(**A**−**E**) The expression of FKBP5 (**A**), IGFBP2 (**B**), PTGER2 (**C**), SAMHD1 (**D**) and TMTC1 (**E**) quantified by qPCR in the uninduced and the osteogenic induced of ADSCs. (**F**) The expression of the biomarkers analyzed by Western blot. (**G**−**K**) Relative intensity analyses of Western blot results of FKBP5 (**G**), IGFBP2 (**H**), PTGER2 (**I**), SAMHD1 (**J**) and TMTC1 (**K**). *p* < 0.001 (“***”).

Given that the majority of the datasets used in our bioinformatic analyses were based on clinically collected adipose derived stem cells (ADSCs), it was only fitting that we also used human ADSCs for our experimental validation. Using in vitro experiments, we looked to verify the expression of the five hub biomarkers in osteogenic ADSCs cell lines. ADSCs were cultured under osteogenic inducing conditions. After 21 days, osteogenic differentiation of ADSCs was confirmed by alizarin red staining (Figure 12A). Red staining of the calcium nodules indicated that ADSCs had been successfully osteogenically induced into osteoblasts. Furthermore, mRNA and protein levels of osteogenesis markers ALP and RUNX2 were significantly up-regulated during the process of osteogenesis (Figure 12B–F), verifying the successful induction of osteogenesis. Using RT-qPCR and Western blot (Figure 13), we found that FKBP5, IGFBP2, PTGER2, SAMHD1, and TMTC1 were all significantly up-regulated in osteogenic induced ADSCs, compared with the normal uninduced ADSCs, with elevated expression levels persisting after osteogenesis induction. This is consistent with the results from our bioinformatic analyses and verifies the efficacy of the biomarkers identified by the latter.

## 3. Discussion

The importance of MSCs in bone tissue engineering has been established. However, their efficacy in osteogenic differentiation is limited without additional in vivo and in vitro investigation [16,17]. Thus, focus has shifted to determining ways in which to improve the efficiency of MSCs in damage restoration. Identifying novel biomarkers of osteogenic induction expressed in stem cells will help clarify both the osteogenic potential of stem cells and the underlying mechanisms by which they act. Being able to stimulate cell differentiation into appropriate bone filling material should increase the proficiency and biocompatibility of bone regeneration, which has obvious clinical benefits. 

In this study, we identified hub biomarkers of stem cell osteogenic induction using a comprehensive strategy of LASSO logistic regression and ROC curve analysis. The five hub biomarkers identified were: FKBP5, IGFBP2, PTGER2, SAMHD1, and TMTC1. Previous bioinformatic analyses have focused on investigating stem-cell-related osteogenic biomarkers [18,19], with our study building on this research. Our study indicated that, with an average AUC > 0.8 for the validation datasets, SAMHD1 and FKBP5 are reliable predictive markers.

Of the five hub biomarkers identified, the effects of IGFBP2 and PTGER2 on osteogenesis have already been reported but remain controversial. IGFBP2, an important member of the insulin-like growth factor family, plays a crucial role in the human body with regards to growth, development, and metabolism [20,21]. Hamidouche et al., demonstrated that the action of IGFBP2 includes the promotion of the osteogenic differentiation of MSCs by enhancing the osteoblast phenotype genes (including RUNX2, ALP, and COI1A1B) [22]. Consistent with our results, the expression of IGFBP2 increased significantly when osteogenesis was induced [23]. Moreover, a recent study revealed that IGFBP2 is also capable of activating the JNK/Akt pathway and inducing adipogenic differentiation of MSCs [24]. Further research is needed to elucidate the mechanism of and the specific roles played by IGFBP2 in promoting osteogenesis/adipogenesis in MSCs. PTGER2 acts as the main receptor of prostaglandin E2, which has been shown to encourage osteogenic differentiation of MSCs and to stimulate the resorption and formation of bones [25,26,27]. Recent studies suggest that local application of PTGER2 agonists to fractured rat bones can effectively stimulate the formation of bone marrow and periosteum, thus promoting fracture repairing [28,29,30]. However, the direct effect of PTGER2 on the osteogenic differentiation of MSCs needs further research/validation.

TMTC1, a glycosyltransferase, promotes O-mannosylation of cadherin and maintains intracellular calcium homeostasis [31,32,33]. Glycosylation is a key post-translational modification involved in controlling extracellular matrix formation during osteogenesis [34,35]. GO analysis revealed that DEGs were mainly concentrated in the extracellular matrix organization and the collagen-containing extracellular matrix. Previous studies have revealed that the extracellular matrix modulates osteogenic effects by adhering to cytoskeletal proteins in MSCs [36,37]. Our results support the possibility that TMTC1 and glycosylation play crucial roles in extracellular matrix formation during osteogenic differentiation.

The close relationship between stem cell osteogenic induction and immunity has been well documented, and a controlled inflammatory response vital for osteogenesis [38,39,40]. In this study, we compared osteogenic induced and uninduced groups to identify differentially expressed immunity and inflammatory-related regulators and numerous DEGs, demonstrating a significant difference in immunity and inflammation modulation between osteogenic and non-osteogenic status. We further analyzed their association with hub biomarkers. Of the five biomarkers, SAMHD1 and FKBP5 were shown to be most correlated with immunity-related and inflammation-related genes during osteogenic differentiation. SAMHD1 is a deoxynucleoside triphosphohydrolase that can suppress the innate immune response to viral infection by interacting with various key proteins in the immune signaling pathways [41,42]. FKBP5 is a stress-responsive molecule that also possesses the ability to modulate immune function. Earlier research has demonstrated the effects of SAMHD1 and FKBP5 on immune response and inflammation, but whether these effects present during the process of osteogenic induction have not been fully elucidated [43]. Our present results suggest that the potential mechanisms of SAMHD1 and FKBP5 in osteogenic induction might be closely linked to immunity and inflammation regulation. 

In summary, we combined bioinformatic and ROC analyses to screen for biomarkers associated with osteogenic induction MSCs and subsequently analyze their potential roles in immunity and inflammation during this process. Additional results from RT-qPCR and Western blots further supported our finding that the expressions of the five biomarkers were all significantly up-regulated during osteogenesis. Although these hub genes are closely associated with the osteogenic induction process of stem cells, and despite this and previous studies (detailed above), the underlying biological mechanisms of osteogenesis remain unclear. Therefore, future research should focus on elucidating the precise mechanisms, as well as discovering novel related signaling pathways and bioactive molecules.

## 4. Materials and Methods

### 4.1. Software and R Packages

R version 3.6.1 software was applied on a Windows platform in this work (URL: https://www.r-project.org/, accessed on 12 January 2020). R packages are open source and downloaded from Bioconductor (URL: http://www.bioconductor.org/, accessed on 30 September 2021). Strawberry Perl version 5.14.2.1 (64-bit) was used to merge the dataset with the merge script in this work (URL: https://www.perl.org/, accessed on 30 September 2021). All software is open source and can be easily accessed via the URLs.

### 4.2. Data Collection and Processing 

Three datasets with expression profiling by high throughput sequencing (GSE159137, GSE159138, GSE114117) and four datasets with expression profiling by the array (GSE88865, GSE153829, GSE63754, GSE73087) were retrieved from the GEO database (http://www.ncbi.nlm.nih.gov/geo, accessed on 3 October 2021), and the characteristics of these included datasets were presented in Supplementary Table S3. The three high throughput sequencing were selected as the train group containing a total of 7 uninduced MSCs samples and 12 osteogenic induced MSCs samples. In addition, four datasets with expression profiling by microarray, with a total of 10 uninduced MSCs samples and 18 osteogenic MSCs induced samples, were utilized for test validation. The RNA-seq and microarray results were combined into a matrix using Perl. The RNA algorithm was applied to background correction and data normalization [44]. Then, probes were transmuted into their corresponding gene symbols based on platform annotation information.

### 4.3. Identification of Differentially Expressed Genes

The analysis of differentially expressed genes (DEGs) was performed by using the “limma” R package (http://www.bioconductor.org/packages/release/bioc/html/limma.html, accessed on 3 October 2021). Adjusted *p*-values were applied to correct the false positive results using the default Benjamini–Hochberg false discovery rate (FDR) method. FDR < 0.05 and |fold change (FC)| > 2 were considered the cutoff values for determining DEGs. “Pheatmap” R package (https://cran.r-project.org/web/packages/pheatmap/index.html, accessed on 4 October 2021), “ggplot2” (https://www.rdocumentation.org/packages/ggplot2/versions/2.1.0, accessed on 4 October 2021) R package and “ggrepel” R package (https://cloud.r-project.org/package = ggrepel, accessed on 4 October 2021) were utilized to visualize DEGs.

### 4.4. Enrichment Analyses of DEGs

Gene Ontology (GO) (http://geneontology.org/, accessed on 10 October 2021) and the Kyoto Encyclopedia of Genes and Genomes (KEGG) (https://www.kegg.jp/, accessed on 10 October 2021) were used for functional and pathway enrichment analyses, respectively. They were performed on the DEGs by using the clusterProfiler package in R. Disease Ontology (DO) (http://disease-ontology.org, accessed on 10 October 2021) comprising 8043 inherited, acquired, and developmental human diseases. Based on the molecular signature database, Gene Set Enrichment Analysis (GSEA) (http://software.broadinstitute.org/gsea/index.jsp, accessed on 10 October 2021) is a statistical tool for interpreting gene expression data. GSEA 4.1.0 was utilized to predict the potential functions and downstream access of the two clusters. Before enrichment analyses, “org.Hs.eg.db” R package (http://www.bioconductor.org/packages/release/data/annotation/html/org.Hs.eg.db.html, accessed on 12 October 2021), a human genome annotation database, was used for the conversion of gene symbol codes into Entrez ID. FDR *q*-value < 0.05 were considered markedly enriched for GO terms, DO, and GSEA analyses, while *p*-value < 0.05 was considered significant for KEGG analyses.

### 4.5. Screening for MSCs Osteogenesis-Related Biomarkers 

LASSO was used to investigate osteogenesis-related biomarkers via the R package “glmnet” R package (https://mirrors.sjtug.sjtu.edu.cn/cran/web/packages/glmnetSE/index.html, accessed on 15 October 2021) in the train group, while the “pROC” R package (https://cran.rstudio.com/web/packages/pROC/index.html, accessed on 15 October 2021) was used for computing the area under the curve (AUC) in the train and the test group. Accuracy, sensitivity, and specificity of a receiver operating characteristic (ROC) curve to assess the diagnostic ability of the markers. *p* < 0.05 was the cut-off for significance.

### 4.6. Selection of Immunity and Inflammation-Related Genes

To make the research more comprehensive, we selected immunity and inflammation-related genes, and the differential expressed genes in osteogenesis and non-osteogenesis groups were visualized by using the methods mentioned above. Immune-related genes were obtained from both https://www.immport.org/home, accessed on 15 October 2021 and https://www.innatedb.ca/, accessed on 15 October 2021 and integrated, and inflammation-related genes were downloaded from https://www.gsea-msigdb.org/gsea/index.jsp, accessed on 15 October 2021. The same method as described above was used to screen for DEGs, and enrichment analysis was performed by GO and KEGG. PPI networks were established to assess the associations among genes in selected modules using the Search Tool for the Retrieval of Interacting Genes version 11 (STRING V11, https://string-preview.org/, accessed on 16 October 2021) with >0.4 as the confidence level. Hub genes, which are presented by highly interconnected nodes, may play vital roles in the PPI network.

### 4.7. Interaction Analysis of Selected Biomarkers

Co-expression analysis of selected biomarkers with immunity-related genes and inflammation-related genes was performed using “limma” in R (http://www.bioconductor.org/packages/release/bioc/html/limma.html, accessed on 20 October 2021), respectively. We then performed co-expression analysis, and the following parameters were used as filter conditions to select related genes: “correlation coefficient = 0.4” and “*p*-value filter = 0.05”.

### 4.8. Human MSCs Culture

Human MSCs, which had been frozen at passage 0, were bought from HyCyte™, Suzhou, China. All donors were exempted from providing informed consent, as the patient specimens used in the study were all discarded specimens after routine clinical diagnosis and privacy-related information was removed. Vials were thawed in a water bath at 37 °C for 1–2 min and thereafter diluted with 10% FBS-supplemented DMEM medium with 1% antibiotic added dropwise for the removal of the cryoprotectant agent. After centrifugation of the sample for 5 min at 1000 r/min, the obtained pellet was resuspended in DMEM and thereafter cultured at 37 °C in a 5% humidified CO_2_ environment.

### 4.9. Osteogenic Differentiation

Passage 2 ADSCs were harvested and seeded in 12 well plates (2 × 10^4^ cells/cm^2^) and incubated to 90% confluence. Then, the conventional growth medium was replaced with osteogenic differentiation medium; 100 mL osteogenic differentiation medium contains 10 mL fetal bovine serum, 1 mL 100 U/mL penicillin and streptomycin (Gibco, Thermo Fisher Scientific, Waltham, MA, USA), 10 μL 10^−8^ mol/L dexamethasone, 1 mL 0.1 mmol/L L-ascorbic acid phosphate (Beyotime, Shanghai, China), 1 mL 2 mmol/L Glutamine (Beyotime, China) and Sodium β-glycerophosphate. Alpha MEM was applied (Hyclone, Logan, UT, USA) to up 100 mL. The medium was renewed every 3 days till day 21. ADSCs were fixed with 4% formaldehyde for 60 min and thereafter stained with 2% alizarin red to observe the effect of osteogenic staining.

### 4.10. Real-Time PCR

RNA extraction was performed using the RNAiso Plus kit (TAKARA, Osaka, Japan), as instructed by the manufacturer. RNA level and purity were determined using an ultra-micro ultraviolet spectrophotometer (IMPLEN-N50; Thermo, Waltham, Massachusetts, USA). A GoScript™ Reverse Transcription kit (Promega, Madison, WI, USA) was used for first-strand cDNA synthesis, while the SystemGoTaq^®^ qPCR Master Mix (Promega, USA) was used in real-time PCR assays on QuantShudio™ real-time PCR instrument (Applied Biosystems, Waltham, MA, USA) to assay the expression levels of RUNX2, ALP, FKBP5, IGFBP2, PTGER2, SAMHD1, and TMTC1. Primer sequences are given in Supplementary Table S4. An unpaired T-test was used to test the significance of observed differences among the study groups.

### 4.11. Western Blot

Protein sample solutions were prepared from ADSCs. The protein concentration was determined using the BCA Protein Assay kit (Beyotime, China). Separating glue of different concentrations and 5% stacking glue separately were prepared and then added to the glass plate. The electrophoresis solution already prepared was poured into the electrophoresis tank, and 5 µg of protein samples were loaded onto the SDS-PAGE gels. Electrophoresis was performed (stacking glue voltage: 80 V, 30 min; the separating glue: 120 V, 60 min), and then proteins were transferred onto PVDF membranes (220 mA, 60 min). The membranes were then blocked and probed with primary antibodies (Supplementary Table S5) overnight at 4 °C, followed by the secondary antibodies (Supplementary Table S5) for 1 h at room temperature. Target protein expression was detected by ECL luminescent solution (Millipore, Boston, Massachusetts, USA) and the Tanon-5500 chemiluminescence imaging analysis system (Tanon, Shanghai, China). An unpaired T-test was used to test the significance of observed differences among the study groups.

## 5. Conclusions

In conclusion, by combining bioinformatics and other analyses, this study has identified several hub biomarkers that are shown to be reliable indicators of osteogenic differentiation of mesenchymal stem cells. Furthermore, we analyzed their potential roles in immunomodulation and inflammatory responses during this process. Our results lay a foundation for us to further study the mechanism of MSCs osteogenic induction.

## Data Availability

Data is contained within the article and Supplementary Material.

## Supplementary Materials

The following supporting information can be downloaded at: https://www.mdpi.com/article/10.3390/ph15091094/s1, Table S1: Co-expression of biomarkers and immune related genes; Table S2: Co-expression of biomarkers and inflammation related genes; Table S3: Gene expression data from Gene Expression Omnibus (GEO) database; Table S4: Primer information; Table S5: Antibody information.
