# Supplementary material for: Identification of Biomarkers That Modulate Osteogenic Differentiation in Mesenchymal Stem Cells Related to Inflammation and Immunity: A Bioinformatics-Based Comprehensive Study
Source: Pharmaceuticals (Basel). 2022 Aug 31;15(9):1094. doi: 10.3390/ph15091094 (PMC9504288; doi:10.3390/ph15091094)
Supplement: Supplementary file 1 [file pharmaceuticals-15-01094-s001.zip › ST1.pdf]

Supplementary table S1. Co-expression of biomarkers and immune related genes.

Immune related gene	Biomarker	cor	*p* value	Regulation
SAMHD1	FKBP5	0.878497	0.000169	positive
APOD	FKBP5	0.841998	0.000591	positive
SAA1	FKBP5	0.657604	0.020119	positive
TSC22D3	FKBP5	0.632426	0.027332	positive
ANGPT1	FKBP5	0.698622	0.011488	positive
PTK2B	FKBP5	0.784954	0.002492	positive
ANOS1	FKBP5	-0.71911	0.008395	negative
NRG1	FKBP5	-0.61721	0.032506	negative
AGTR1	FKBP5	0.791893	0.002142	positive
EDNRB	FKBP5	0.612917	0.034084	positive
PDGFD	FKBP5	0.728478	0.00721	positive
SDC1	FKBP5	-0.60023	0.039067	negative
C7	FKBP5	0.679423	0.015088	positive
C5AR2	FKBP5	0.645377	0.023422	positive
GDF7	FKBP5	0.65612	0.0205	positive
EPGN	FKBP5	-0.65542	0.020681	negative
A2M	FKBP5	0.645669	0.023339	positive
UCN2	FKBP5	0.678846	0.015208	positive
PTH1R	FKBP5	0.577018	0.049494	positive
NGFR	FKBP5	0.746981	0.005242	positive
SAMHD1	IGFBP2	0.638202	0.025534	positive
APOD	IGFBP2	0.812225	0.001329	positive
LGR4	IGFBP2	-0.77284	0.003206	negative
ANGPTL4	IGFBP2	0.733831	0.006592	positive
PTK2B	IGFBP2	0.645715	0.023325	positive
NRG1	IGFBP2	-0.65704	0.020262	negative
AGTR1	IGFBP2	0.781886	0.00266	positive
PDGFD	IGFBP2	0.611956	0.034444	positive
LEP	IGFBP2	0.740169	0.005912	positive
SDC1	IGFBP2	-0.77122	0.003313	negative
NR2F1	IGFBP2	0.718353	0.008497	positive
C7	IGFBP2	0.814873	0.001244	positive
EPGN	IGFBP2	-0.87914	0.000165	negative
STC1	IGFBP2	0.635467	0.026375	positive
A2M	IGFBP2	0.703206	0.010733	positive
CCL13	IGFBP2	0.701208	0.011058	positive
UCN2	IGFBP2	0.816073	0.001206	positive
PTH1R	IGFBP2	0.645509	0.023384	positive
NGFR	IGFBP2	0.703752	0.010645	positive
SAMHD1	SAMHD1	1	1.03E-70	positive

APOD	SAMHD1	0.813539	0.001286	positive
PTGER2	SAMHD1	0.586136	0.04519	positive
SAA1	SAMHD1	0.698088	0.011579	positive
MMP7	SAMHD1	0.597863	0.040052	positive
TSC22D3	SAMHD1	0.652469	0.021461	positive
ANGPT1	SAMHD1	0.626446	0.029289	positive
PTK2B	SAMHD1	0.653912	0.021078	positive
ANOS1	SAMHD1	-0.7379	0.006149	negative
AGTR1	SAMHD1	0.706966	0.010141	positive
GDF7	SAMHD1	0.661101	0.01924	positive
EPGN	SAMHD1	-0.65035	0.022035	negative
SLC40A1	SAMHD1	0.679409	0.015091	positive
A2M	SAMHD1	0.616625	0.032717	positive
NGFR	SAMHD1	0.686714	0.013635	positive
IL6	SAMHD1	-0.57936	0.04836	negative
SAMHD1	TMTC1	0.82189	0.001038	positive
PTGER2	TMTC1	0.822676	0.001017	positive
LGR4	TMTC1	-0.65592	0.020552	negative
ANGPT1	TMTC1	0.700543	0.011167	positive
LEPR	TMTC1	0.582698	0.046781	positive
ANOS1	TMTC1	-0.79171	0.002151	negative
NRG1	TMTC1	-0.63645	0.026069	negative
EDNRB	TMTC1	0.617272	0.032483	positive
C5AR1	TMTC1	0.71421	0.00907	positive
C5AR2	TMTC1	0.639859	0.025034	positive
IL1RL1	TMTC1	0.650512	0.02199	positive
GDF7	TMTC1	0.840359	0.000621	positive
SLC40A1	TMTC1	0.687872	0.013415	positive
PPARGC1A	TMTC1	0.665063	0.018279	positive
AREG	TMTC1	0.6712	0.01686	positive
CRABP2	TMTC1	-0.59813	0.039942	negative
SAMHD1	PTGER2	0.586136	0.04519	positive
PTGER2	PTGER2	1	7.94E-69	positive
ANOS1	PTGER2	-0.70111	0.011074	negative
SLC40A1	PTGER2	0.630415	0.027979	positive
PPARGC1A	PTGER2	0.80576	0.001556	positive
CRABP2	PTGER2	-0.80215	0.001694	negative
IL6	PTGER2	-0.71855	0.00847	negative
