# Supplementary material for: Identification of Biomarkers That Modulate Osteogenic Differentiation in Mesenchymal Stem Cells Related to Inflammation and Immunity: A Bioinformatics-Based Comprehensive Study
Source: Pharmaceuticals (Basel). 2022 Aug 31;15(9):1094. doi: 10.3390/ph15091094 (PMC9504288; doi:10.3390/ph15091094)
Supplement: Supplementary file 1 [file pharmaceuticals-15-01094-s001.zip › ST2.pdf]

Supplementary table S2. Co-expression of biomarkers and inflammation related genes.

Inflammation related gene	Biomarker	cor	*p* value	Regulation
BDKRB1	FKBP5	-0.65123958	0.021792337	negative
CD82	FKBP5	0.863575418	0.00029466	positive
CDKN1A	FKBP5	-0.782368639	0.002633147	negative
CHST2	FKBP5	0.659470315	0.019646225	positive
CXCR6	FKBP5	0.673761791	0.016292707	positive
INHBA	FKBP5	-0.721062794	0.00813693	negative
LPAR1	FKBP5	0.649708006	0.022209777	positive
MXD1	FKBP5	-0.680800725	0.014805463	negative
PTGER4	FKBP5	0.70652552	0.010209257	positive
RIPK2	FKBP5	-0.632218742	0.027398517	negative
SLC4A4	FKBP5	-0.623010993	0.030456877	negative
TNFSF9	FKBP5	-0.58604887	0.045229839	negative
ADM	IGFBP2	0.619614804	0.03164453	positive
CD40	IGFBP2	0.637875106	0.025633574	positive
CD82	IGFBP2	0.905790243	0.0000498	positive
CDKN1A	IGFBP2	-0.646818915	0.023013017	negative
CHST2	IGFBP2	0.872735878	0.000211527	positive
IFITM1	IGFBP2	0.757047546	0.00435922	positive
INHBA	IGFBP2	-0.749747119	0.004987399	negative
LPAR1	IGFBP2	0.593798189	0.041783501	positive
MXD1	IGFBP2	-0.794320094	0.002028742	negative
PTGER4	IGFBP2	0.788471206	0.002309513	positive
RIPK2	IGFBP2	-0.719995108	0.008277386	negative
SLC4A4	IGFBP2	-0.745148832	0.005416701	negative
TLR2	IGFBP2	0.604987195	0.037142038	positive
TNFRSF1B	IGFBP2	0.728583385	0.007197474	positive
BDKRB1	SAMHD1	-0.722948357	0.007893244	negative
CD82	SAMHD1	0.752408175	0.004750999	positive
CDKN1A	SAMHD1	-0.604274036	0.037426353	negative
CXCR6	SAMHD1	0.639525823	0.02513441	positive
IL1R1	SAMHD1	0.587393356	0.044618005	positive
IL6	SAMHD1	-0.579363545	0.048360329	negative
PTGER2	SAMHD1	0.586135805	0.045190099	positive
RIPK2	SAMHD1	-0.655653831	0.020621179	negative
SCN1B	SAMHD1	-0.636717191	0.025987976	negative
SLC4A4	SAMHD1	-0.582409884	0.046915516	negative
C5AR1	TMTC1	0.714209971	0.009070195	positive
CD82	TMTC1	0.721169657	0.00812297	positive
CXCR6	TMTC1	0.833635546	0.000753658	positive

LPAR1	TMTC1	0.608111384	0.035914653	positive
MXD1	TMTC1	-0.63942472	0.025164779	negative
PTGER2	TMTC1	0.822676097	0.001016776	positive
TNFAIP6	TMTC1	-0.582544685	0.046852295	negative
TNFSF9	TMTC1	-0.677741891	0.015438898	negative
CXCR6	PTGER2	0.69943985	0.011350871	positive
FZD5	PTGER2	0.603262432	0.037832306	positive
IL6	PTGER2	-0.718552304	0.00847006	negative
PTGER2	PTGER2	1	5.71E-66	positive
RAF1	PTGER2	0.656214723	0.020475727	positive
TNFAIP6	PTGER2	-0.586483484	0.045031415	negative
TNFSF9	PTGER2	-0.63902551	0.02528495	negative
