# Supplementary material for: Identification of Biomarkers That Modulate Osteogenic Differentiation in Mesenchymal Stem Cells Related to Inflammation and Immunity: A Bioinformatics-Based Comprehensive Study
Source: Pharmaceuticals (Basel). 2022 Aug 31;15(9):1094. doi: 10.3390/ph15091094 (PMC9504288; doi:10.3390/ph15091094)
Supplement: Supplementary file 1 [file pharmaceuticals-15-01094-s001.zip › ST3.pdf]

Supplementary table S3. Gene expression data from Gene Expression Omnibus (GEO) database.

Dataset ID	Total samples	Control	Treat	Data type	Platforms	Tissue type	Country	Species	Source
GSE159137	6	6	0	RNA-seq	Illumina HiSeq 2000	ADSCs	Germany	Homo sapiens	**Plastic surgery residual material**
GSE159138	6	0	6	RNA-seq	Illumina HiSeq 2000	ADSCs	Germany	Homo sapiens	**Plastic surgery residual material**
GSE114117	7	1	6	RNA-seq	Illumina HiSeq X Ten	BMSCs	China	Homo sapiens	**Donation**
GSE88865	6	3	3	Microarray	Affymetrix	PDMSCs	China	Homo sapiens	**Donation**
GSE153829	6	0	6	Microarray	Agilent	BMSCs	China	Homo sapiens	**Donation**
GSE63754	6	3	3	Microarray	Agilent	ADSCs	Lithuania	Homo sapiens	**Plastic surgery residual material**
GSE73087	7	1	6	Microarray	Affymetrix	BMSCs	China	Homo sapiens	**Purchased from LONZA**
