# Supplementary material for: Identification of Biomarkers That Modulate Osteogenic Differentiation in Mesenchymal Stem Cells Related to Inflammation and Immunity: A Bioinformatics-Based Comprehensive Study
Source: Pharmaceuticals (Basel). 2022 Aug 31;15(9):1094. doi: 10.3390/ph15091094 (PMC9504288; doi:10.3390/ph15091094)
Supplement: Supplementary file 1 [file pharmaceuticals-15-01094-s001.zip › ST4.pdf]

Supplementary table S4. Primer information

Primer name	Primer sequences (5'-3')
RUNX2-F	ATCTCCGCAGGTCACTACCA
RUNX2-R	ACTGTGCTGAAGAGGCTGTTT
ALP-F	TCCTGTTGACACCCCAAACC
ALP-R	CACATGCCCATGCAACACTT
FKBP5-F	AAATTCCTCGCTGCCGGACT
FKBP5-R	CCTCTGAGAAGCGTTCTGTCC
IGFBP2-F	CACTTGTGAGAAGCGCCG
IGFBP2-R	TGAGTGGTCATCGCCATTGT
PTGER2-F	GCAGTCTCCCTGCTCTTCTG
PTGER2-R	CACCGAGACAATGAGAAGCA
SAMHD1-F	ACAGACTCACAAGCACCTGG
SAMHD1-R	TACTGTAGCAGCGTCGTTCC
TMTC1-F	TGTGGGCGATCGTGAACAAC
TMTC1-R	TAGCTTGAAGGTGAGGACGC
hGAPDH-F	TCAAGGCTGAGAACGGGAAG
hGAPDH-R	TGGACTCCACGACGTACTCA
