# Supplementary material for: Identification of Biomarkers That Modulate Osteogenic Differentiation in Mesenchymal Stem Cells Related to Inflammation and Immunity: A Bioinformatics-Based Comprehensive Study
Source: Pharmaceuticals (Basel). 2022 Aug 31;15(9):1094. doi: 10.3390/ph15091094 (PMC9504288; doi:10.3390/ph15091094)
Supplement: Supplementary file 1 [file pharmaceuticals-15-01094-s001.zip › ST5.pdf]

Supplementary table S5. Antibody information

Antibody name	Product Code	Manufacturer	Country
ALP	ABP53294	Abbkine	China
RUNX2	ABP53087	Abbkine	China
FKBP5	14155-1-AP	proteintech	USA
IGFBP2	11065-3-AP	proteintech	USA
PTGER2	38496-1	Signalway	China
SAMHD1	12586-1-AP	proteintech	USA
TMTC1	23349-1-AP	proteintech	USA
GAPDH	TA-08(1:1000)	ZSGB-BIO	China
Goat anti-mouse secondary antibody	31430 (1:10000)	Thermo	USA
Goat anti-rabbit secondary antibody	31460 (1:10000)	Thermo	USA
